# Estimation of the Burden of Disease Due to Diabetes Mellitus Type 2 in the Population of Tabasco During the Period 2013–2023

**DOI:** 10.3390/ijerph22070997

**Published:** 2025-06-24

**Authors:** David Ricardo Hernández-Bartolo, Sergio Quiroz-Gomez, Crystell Guadalupe Guzmán-Priego, Karla del Socorro Celorio-Méndez, Isela Esther Juárez-Rojop, Jorge Luis Ble Castillo, Marisol Guzmán-Moreno, Sergio de Jesús Romero Tapia, Alejandro Jiménez-Sastré, Sonia Martha López-Villarreal, Osvelia E. Rodríguez-Luis, Laura Elena Villarreal-García

**Affiliations:** 1 Health Sciences Academic Division (DACS), Juarez Autonomous University of Tabasco (UJAT), Villahermosa 86040, Mexico; 182e45304@egresados.ujat.mx (D.R.H.-B.); karla.celorio@ujat.mx (K.d.S.C.-M.); sergio.romero@ujat.mx (S.d.J.R.T.); alejandro.jimenez@ujat.mx (A.J.-S.); 2 Cardiometabolism Laboratory, Research Center, Health Sciences Academic Division (DACS), Juarez Autonomous University of Tabasco (UJAT), Villahermosa 86040, Mexico; crystell.guzman@ujat.mx; 3 Lipid Metabolism Laboratory, Research Center, Health Sciences Academic Division (DACS), Juarez Autonomous University of Tabasco (UJAT), Villahermosa 86040, Mexico; isela.juarez@ujat.mx; 4 Metabolic Diseases Biochemistry Laboratory, Research Center, Health Sciences Academic Division (DACS), Juarez Autonomous University of Tabasco (UJAT), Villahermosa 86040, Mexico; jorge.ble@ujat.mx; 5 Multidisciplinary Academic Division of Los Rios, Juarez Autonomous University of Tabasco (UJAT), Tenosique 86901, Mexico; marisol.guzman@ujat.mx; 6 Research Department, Autonomous University of Nuevo Leon, Monterrey 64460, Mexico; sonia.lopezvl@uanl.edu.mx (S.M.L.-V.); osvelia.rodriguezls@uanl.edu.mx (O.E.R.-L.); laura.villarrealgr@uanl.edu.mx (L.E.V.-G.)

**Keywords:** people with disabilities, diabetes complications, epidemiologic research design, environment and public health, epidemiologic factors, cohort studies, mortality, cost of illness

## Abstract

Background: The burden of type 2 diabetes mellitus (T2DM) in Tabasco from 2013 to 2023 has led to a significant loss in quality of life and life years. This study aims to analyze the impact of T2DM on the population of Tabasco, Mexico, during this period. Methods: A descriptive, observational, longitudinal, and retrospective study was conducted in Tabasco, following Feinstein’s guidelines. The study included 2,402,598 individuals, covering the adult study population of Tabasco (*n* = 927,047) based on National Institute of Statistics and Geography (INEGI) data. Data were gathered from the General Directorate of Health Information and the Ministry of Health and analyzed using Microsoft Excel, applying central tendency and dispersion measures, and calculating Disability-Adjusted Life Years (DALYs) with validated formulas. Results: DALYs in Tabasco’s adult population from 2013 to 2023 were 23,049 in 2013, 24,576 in 2014, 25,193 in 2015, 34,361 in 2016, 29,771 in 2017, 29,309 in 2018, 29,959 in 2019, 28,087 in 2020, 26,451 in 2021, 23,502 in 2022, and 30,523 in 2023, totaling 304,781 DALYs for the period. Conclusions: T2DM has shown an increase in incidence and mortality, especially in recent years, leading to a significant rise in DALYs. This reflects a higher disease burden in Tabasco compared to other regions in Mexico and the Americas, resulting in a considerable loss of quality of life and life years.

## 1. Introduction

Type 2 diabetes mellitus (T2DM) has shown a significant upward trend in prevalence and incidence, becoming one of the leading causes of death and disability worldwide. Currently, 536 million people suffer from diabetes, and it is projected that this figure will increase to 783 million by 2045. In Mexico, prevalence reached 16.8% in 2018, ranking as the second leading cause of death and leading cause of disability in the country. Despite some improvements in detection and stabilization of incidence rates in some regions, the burden of disease remains a critical public health challenge [1]. In 2021, diabetes was responsible for 13% of deaths in Mexico, equivalent to 140,729 deaths, with 74.9% of the deceased being non-insulin dependent. The mortality rate for this disease increased from 8.2 deaths per 10,000 inhabitants in 2019 to 11 in 2021. A high percentage of deaths occurred among people not affiliated with health institutions, highlighting the need for effective health policies. Contributing factors such as obesity, physical inactivity, and unhealthy dietary patterns are also highly prevalent in the Mexican population, exacerbating the diabetes epidemic [2,3].

Recent studies in Mexico indicate that the prevalence of diabetes in adults is approximately 18.3%, and 22.1% of the population have prediabetes. Between 2006 and 2022, the prevalence of the disease increased by 3.9%, while the proportion of undiagnosed cases decreased by 1.3%, suggesting improvements in detection [4]. However, some experts caution that focusing on prevalence alone may divert attention from the preventive and lifestyle strategies needed to address the disease comprehensively [5].

T2DM has reached epidemic levels globally, affecting millions of people and generating multiple chronic complications, such as cardiovascular disease, renal failure, and amputations. These problems not only impact quality of life but also health systems, increasing economic costs. Preventable factors such as obesity, physical inactivity, and unhealthy diets contribute to the disease. T2DM especially affects vulnerable and low-income populations, where limited access to health services and education aggravates the situation [6].

In Mexico, T2DM is a leading cause of morbidity and mortality, affecting 10–15% of the adult population. Between 2023 and 2024, there was a 50.6% increase in reported cases. According to the Global Burden of Disease, the incidence rate of diabetes in Mexico increased from 367 to 496 cases per 100,000 inhabitants between 2006 and 2019. This increase is likely multifactorial; current evidence shows that the genetic predisposition to T2DM is driven by over 400 common risk alleles, most notably the TCF7L2 rs7903146 variant. This variant confers an odds ratio (OR) of approximately 1.4 per copy and accounts for around 10% of heritable risk [7]. Aging amplifies susceptibility: Every decade after the age of 40 raises the risk of incident T2DM by ~30%, meaning that adults aged 65 and over have an OR of ~2.3 compared to those aged 40–44 years [8]. Obesity is the dominant modifiable risk factor: A body mass index (BMI) of ≥30 kg/m^2^ multiplies the risk by four to sevenfold, and each 5 kg/m^2^ increase in BMI increases the risk by 1.5-fold (95% confidence interval, 1.4–1.6). Elevated BMI currently explains ~47% of T2DM Disability Adjusted Life Years (DALY) in Mexico [9].

According to INEGI, Tabasco has a diabetes-related mortality rate of 13.5 deaths per 10,000 inhabitants, which is higher than in Chiapas (10.4), Campeche (9.7), and Yucatán (6.3). ENSANUT-2022 reports the highest prevalence of adult obesity (38.2%) and diabetes mellitus type 2 (DM2) (18.5%) in the south-east, compared to national averages of 16.8% and 16.8%, respectively. The density of primary care units in the area is 0.8 per 10,000 inhabitants, compared to a national average of 1.1. Additionally, approximately 66% of the population lives in multidimensional poverty, with 46% residing in rural areas. Regions with comparable socioeconomic features, e.g., Veracruz and Oaxaca, have similar mortality-prevalence gaps, underscoring the importance of structural determinants [10,11,12].

Addressing these gaps requires improving education and prevention at the local level, in addition to conducting research to better evaluate interventions and strategies to reduce the burden of T2DM. Therefore, the health impact of a disease cannot be evaluated using incidence or prevalence figures alone; its overall health burden must also be estimated. The reference indicator is the Disability-Adjusted Life Year (DALY), which combines Years of Life Lost due to premature mortality (YLL) and Years Lived with Disability (YLD) into one metric. Adopted by the World Health Organization and the Global Burden of Disease (GBD) consortium, this measure quantifies ‘healthy lifetime lost’ and enables uniform comparisons of causes, regions, and periods [13,14].

For Mexico, the GBD reports place type 2 diabetes mellitus (DM2) among the top two causes of DALYs, with values close to 1300 per 100,000 inhabitants. However, these estimates are national and mask subnational heterogeneity. None have examined the state of Tabasco in detail, despite it having the highest obesity prevalence in the south-east and excess mortality from DM2 above the national average [15].

This research uses the GBD methodology to estimate the burden of type 2 diabetes (DM2) in adults in Tabasco for the first time, from 2013 to 2023. This approach enables us to accurately evaluate the health impact of DM2 and generate evidence to inform the prioritization and design of prevention and control policies in the state.

## 2. Materials and Methods

### 2.1. Type of Study

This research is framed within an observational, descriptive, longitudinal, and retrospective type of investigation in the population of Tabasco during the period 2013–2023.

### 2.2. Universe

The universe of the study encompasses 2,402,598 people in Tabasco, according to data provided by the National Institute of Statistics Geography and Informatics (INEGI) [11].

### 2.3. Study Population

It consisted of 927,000 inhabitants of legal age, of which 462,000 were men and 465,000 were women [11]. Because administrative and census records include all adults ≥20 years residing in Tabasco, the study analyzed a full-coverage population; therefore, no probabilistic sampling or representativeness weighting was required.

### 2.4. Data Collection Method and Instrument

Data were organized in spreadsheets, and simple arithmetic calculations (addition, subtraction, multiplication, and division) were performed to determine the burden of disease. Information on annual deaths was obtained from the General Directorate of Health Information (DGIS) (http://www.dgis.salud.gob.mx/contenidos/basesdedatos/bdc_defunciones_gobmx.html) (accessed on 6 February 2025) [12]. Data on life expectancy were extracted from the National Institute of Statistics and Geography (INEGI), applying filters by state, year, and sex (https://www.inegi.org.mx/app/tabulados/interactivos/?pxq=Mortalidad_Mortalidad_09_1ac777ca-020d-4a8c-ae90-ec74f60b6ae8) (accessed on 11 March 2025) [13]. The incidence of the disease, disaggregated by age group and state, was obtained from the Morbidity Yearbook for the period 1984–2023, available on the official website of the General Directorate of Epidemiology (https://epidemiologia.salud.gob.mx/anuario/html/incidencia_enfermedad.html) (accessed on 17 April 2025) [14].

### 2.5. Data Analysis

Through content analysis, measures of central tendency and dispersion were carried out, in addition to the calculation of DALYs. Frequency tables were created to present the data in an organized manner, and bar and line graphs were produced to facilitate the visualization of the results [15,16]. Data and formula checks were performed to ensure accuracy, and the results were interpreted in relation to the theoretical framework and study objectives. A sensitivity analysis was performed to evaluate the robustness of the results in response to variations in key variables (mortality and life expectancy).

### 2.6. Calculate DALYs

1. Years of Life Lost (YLL): The total annual deaths were multiplied by life expectancy, calculated as the average for each age group. For individuals over 65, life expectancy was considered 0.0 due to reduced potential lifespan.

YLL = Number of deaths × Standard life expectancy at age of death

2. Years Lived with Disability (YLD): The disease duration was multiplied by the disability weight (from the DALY Calculator) and the incidence rate for each age group to calculate YLD for T2DM.

YLD = Number of prevalent cases × Disability weight

3. Disability-Adjusted Life Years (DALY): YLL and YLD were summed to determine the DALYs.

DALY = YLL + YLD

### 2.7. Inclusion and Exclusion Criteria

#### 2.7.1. Inclusion Criteria

− People over 20 years old with T2DM,
− Non-insulin-dependent diabetes mellitus,
− Diabetes mellitus associated with malnutrition,
− Unspecified diabetes mellitus.

#### 2.7.2. Exclusion Criteria

− Minors,
− Records with incomplete information or missing data that prevent proper analysis,
− Cases reported outside the 2013–2023 period,
− Reported cases of type 1 diabetes (T1DM),
− Insulin-dependent diabetes mellitus,
− Other specified diabetes mellitus.

### 2.8. Ethical Considerations

The research was conducted in accordance with the ethical principles of the Declaration of Helsinki, prioritizing the well-being and rights of the participants over scientific objectives. Although historical data were used, strict ethical protocols were applied for their management. Confidentiality was guaranteed by anonymizing the data, restricting access to the research team, and presenting results in aggregate. The protocol was approved by the Research Committee of the Academic Division of Health Sciences of the Juarez Autonomous University of Tabasco with registration number JI-LCT204, in accordance with NOM-012-SSA3-2012 and General Health Law. As no biological or hazardous agents were handled, biosafety measures centered on protecting information in accordance with current regulations.

## 3. Results

**Figure 1:**
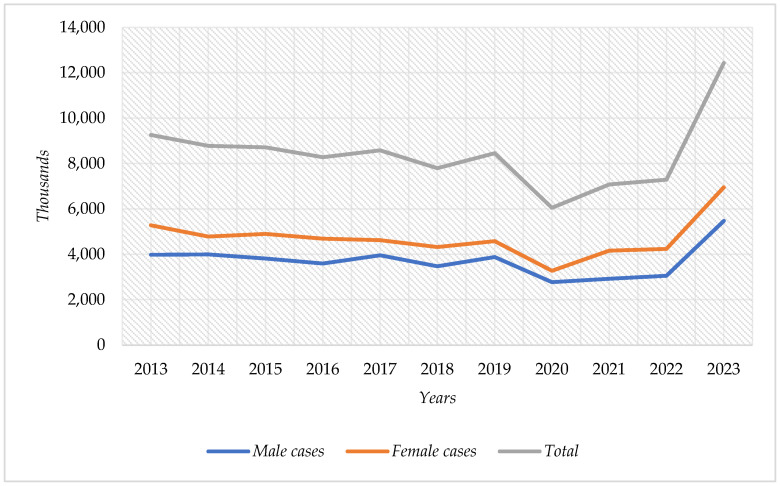
Incidence of T2DM in the Adult Population by Year and Sex in Tabasco, 2013–2023.

The population under study comprised 927,000 inhabitants over 20 years of age in Tabasco, with almost equal distribution by sex (51.1% women and 48.9% men) and a median age of 29 years. From 2013 to 2023, a total of 93,750 cases of T2DM were documented, with 40,909 cases recorded in men and 51,781 cases recorded in women, according to official data from the General Directorate of Health Information (Figure 1).

**Figure 2:**
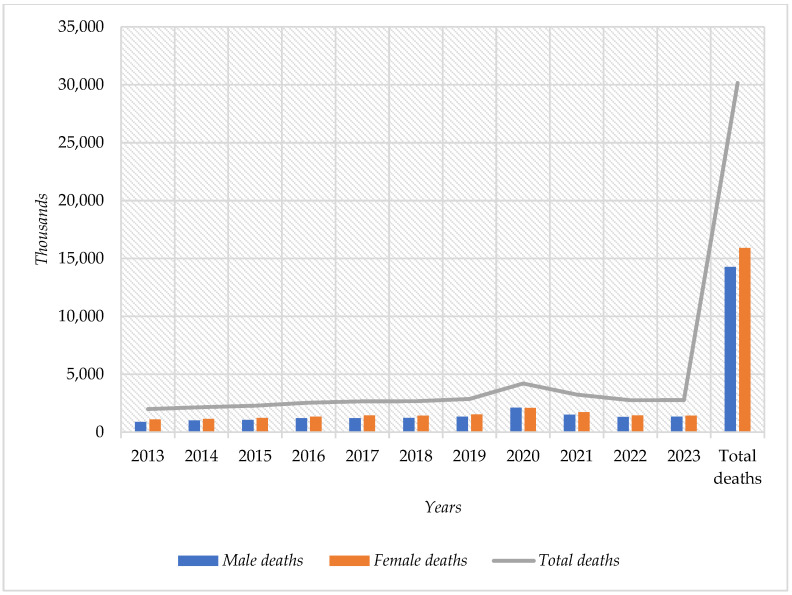
Mortality of T2DM in the Adult Population by Year and Sex in Tabasco, 2013–2023.

From 2013 to 2023, a total of 30,166 deaths due to T2DM were documented in Tabasco, of which 14,268 cases occurred in men and 15,898 cases occurred in women. This data indicates a higher mortality rate in the female population during the period under analysis (Figure 2).

### Disability Adjusted Life Years (DALYs)

From 2013 to 2023, a total of 430,367 DALYs attributable to T2DM were documented in the adult population of Tabasco, with a peak in 2016 and another notable increase in 2023.

**Figure 3:**
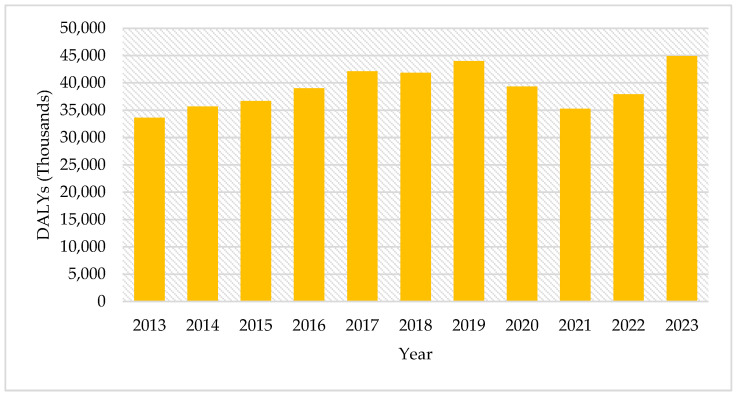
Total DALYs of T2DM in the Adult Population by Year in Tabasco, 2013–2023.

DALYs by year: 33,634 in 2013, 35,686 in 2014, 36,665 in 2015, 38,998 in 2016, 42,135 in 2017, 41,840 in 2018, 44,000 in 2019, 39,335 in 2020, 35,274 in 2021, 37,901 in 2022, and 44,899 in 2023 (Figure 3). Thus, the adult population of Tabasco lost an average of 39,100 healthy life years each year, equivalent to 4220 disability-adjusted life years (DALYs) per 100,000 inhabitants. This highlights a province-wide burden that exceeds the national average for 2021 (approximately 3400 per 100,000).

**Figure 4:**
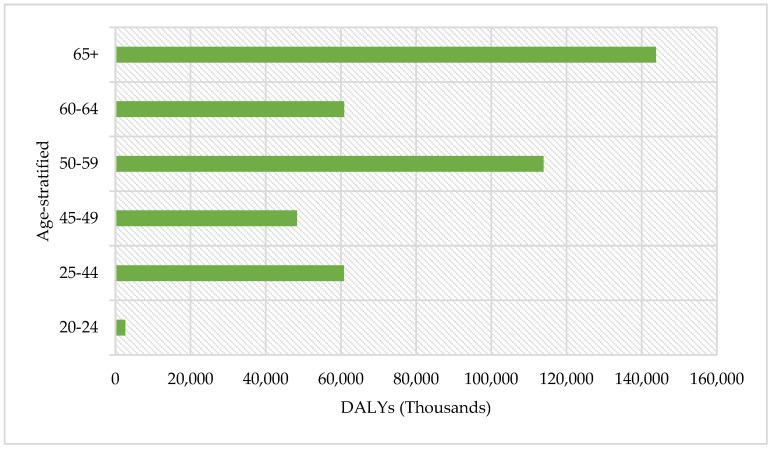
Total DALYs by age group in the period 2013–2023.

Figure 4 reveals a steep age gradient in diabetes burden. Over the 2013–2023 period, the ≥65 y cohort accumulated 150,000 DALYs, or 34% of the total, reflecting high mortality and long-standing complications at advanced ages. The 50–59 y stratum contributes a further 110,000 DALYs (25%), while the adjacent 60–64 y group adds 70,000 DALYs (16%). Thus, three age bands ≥ 50 y account for 75% of all healthy-life years lost in Tabasco, underscoring the combined effect of cumulative hyperglycemic exposure and age-related β-cell decline. Conversely, adults < 45 y generate <10% of DALYs, yet their losses occur during peak productive years.

**Figure 5:**
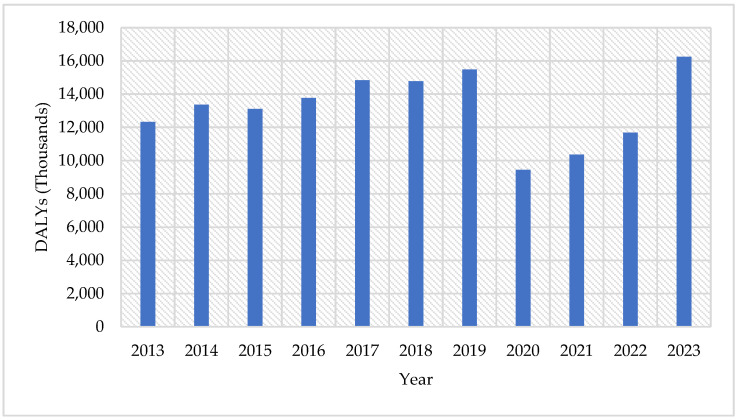
Total DALYs of T2DM in the Adult Men Population by Year in Tabasco, 2013–2023.

During the period 2013–2023, the burden of disease measured in DALYs among adult males in the state of Tabasco exhibited notable fluctuations. The annual DALYs were as follows: 12,332 in 2013; 13,361 in 2014; 13,117 in 2015; 13,774 in 2016; 14,830 in 2017; 14,775 in 2018; 15,485 in 2019; 9,445 in 2020; 10,356 in 2021; 11,685 in 2022; and 16,247 in 2023. Cumulatively, a total of 145,407 DALYs were recorded over the ten-year period, reflecting the evolving epidemiological profile and health challenges faced by this population group. This corresponds to an average loss of 13,200 healthy life years per year, equivalent to 2860 DALYs per 100,000 men annually. In other words, it is as if the male population were to forfeit 36 years of healthy life every day (Figure 5).

**Figure 6:**
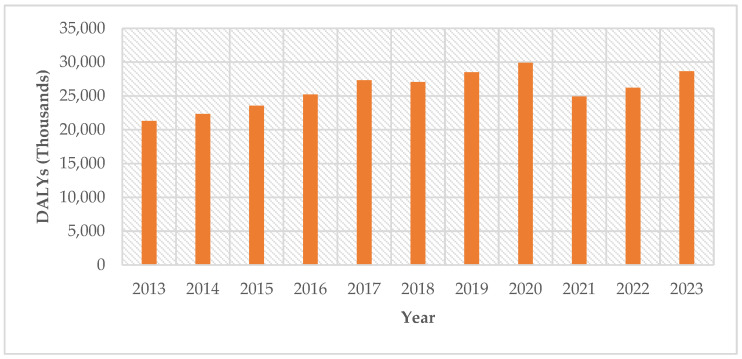
Total DALYs of T2DM in the Adult Women Population by Year in Tabasco, 2013–2023.

During the period from 2013 to 2023, the DALYs among adult females in the state of Tabasco demonstrated considerable variation. The annual DALYs were as follows: 21,302 in 2013; 22,325 in 2014; 23,549 in 2015; 25,223 in 2016; 27,305 in 2017; 27,065 in 2018; 28,515 in 2019; 29,891 in 2020; 24,918 in 2021; 26,215 in 2022; and 28,652 in 2023. Between 2013 and 2023, women in Tabasco lost an average of 25,900 healthy life years per year to type 2 diabetes mellitus (T2DM), equivalent to 284,960 DALYs over the ten-year period. This equates to 2800 DALYs per 100,000 women annually. In practical terms, the female population forfeits approximately 71 years of healthy life every day due to diabetes-related death or disability. This burden is almost double that experienced by men (145,407 DALYs), highlighting a significant gender disparity driven by a higher prevalence of complications and disability among women. (Figure 6).

The sensitivity analysis indicates that a progressive increase in deaths due to T2DM (5%, 10%, 15%, up to 25%) results in a proportional increase in Years of Life Lost (YLL) in both sexes, with a slightly greater impact on women. Conversely, an increase in life expectancy concomitantly leads to an increase in DALYs, as premature deaths in populations with greater longevity engender a greater loss of potential years. The findings indicate that the prevalence of diabetes in Tabasco is particularly responsive to fluctuations in mortality and life expectancy, exhibiting notable disparities between male and female populations. A 5% reduction in mortality could lead to a substantial decrease in DALYs, particularly among the female demographic. However, the simultaneous absence of measures to prevent disability might exacerbate the overall disease burden.

Trend analysis. Annual DALY rates (2013–2023) were examined with Joinpoint log-linear regression (Joinpoint v4.9.1). The permutation test (4499 replicates; α = 0.05) selected a model with two statistically significant Joinpoints, located in 2018 and 2021. The segment-specific annual percentage change (APC) estimates were

2013–2018 APC = +5.7% yr^−1^ (95% CI + 1.9; +9.6, *p* = 0.018).

2018–2021 APC = −6.6% yr^−1^ (95% CI −10.8; −2.2, *p* = 0.022)

2021–2023 APC = +10.7% p.a.^1^ (95% CI + 3.1; +18.7, *p* = 0.014)

The Average Annual Percentage Change (APC) for the entire period was +3.0% yr^−1^ (95% CI + 0.9; +5.1, *p* = 0.011), confirming a net upward trend despite the transitory decline between 2018 and 2021. The result is provided in the Supplementary File.

## 4. Discussion

Between 2013 and 2023, T2DM generated 430,367 DALYs in the adult population of Tabasco, 84% of which were attributable to premature mortality (YLL). The burden increased by an average of +3% per year, with significant changes in 2018 and 2021, and it was almost double the level observed in men. The following sections analyze the epidemiological and structural drivers of these trends, sex-specific differences, alignment with national GBD estimates, and the policy leverage points revealed by the YLL to YLD composition.

The incidence and mortality from T2DM in Mexico, and specifically in the state of Tabasco, have shown a sustained growth in recent years, with a more notable impact on the female population. As demonstrated by the Global Burden of Disease data, there is evidence indicative of an increase in the national incidence rate. This rate rose from 367 to 496 cases per 100,000 inhabitants between 2006 and 2019. This tendency is consistent with the results of the present study [17]. Regarding mortality rates, in 2021, the state of Tabasco registered a rate of 13.5 per 10,000 inhabitants, thus positioning it as the fifth entity with the highest rates at the national level, above adjacent states such as Chiapas, Campeche, and Yucatán [18].

The present study reports an even higher rate of 29.94 deaths per 10,000 inhabitants, with a significant increase from 2020 (46.5% compared to 2019); possibly, the increase in DALYs from 2020 to 2022 may have been multifactorial. Firstly, the pandemic acted as a mortality amplifier for people with T2DM: national vital statistics data show that diabetes was included as a contributing cause in 44% of SARS-CoV-2 deaths in 2021, increasing DALYs by 18% that year. Secondly, the pandemic reduced outpatient services, resulting in poorer metabolic control and an increased number of acute complications, which increased YLD. Thirdly, the catch-up effect of delayed diagnoses at the end of 2021/2022 inflated the recorded incidence and prevalence once services normalized [19]. In 2022, the state of Tabasco registered the highest number of hospital admissions for T2DM, with a total of 5843 cases. This phenomenon could be indicative of a growing trend in the prevalence and mortality of T2DM in Mexico, particularly in the state of Tabasco, which has been observed to exhibit a consistent upward trajectory over recent years, with a more pronounced impact observed on the female demographic [17].

Mexico is the fourth most affected country worldwide in terms of disease burden due to T2DM. In 2021, the disease generated 3.1 million DALYs in the country, representing 6.6% of the total burden, with 64% attributed to premature deaths [9,19]. In Tabasco, the years 2016 and 2023 were pivotal in terms of DALY escalation, exhibiting a 30% surge between 2022 and 2023, likely influenced by post-pandemic repercussions. A detailed analysis of the data reveals a higher disease burden in women, especially among those over 50 years of age. There have been notable increases in the years 2020 to 2022, which coincide with the pandemic. The 50–59 and 65+ age groups consistently present the highest number of DALYs, which is indicative of both populations aging and the vulnerability of these groups to T2DM, according to the data obtained in this study.

The high prevalence of T2DM in Mexico poses a significant challenge to the national healthcare system and adversely impacts both life expectancy and quality of life. Effective disease prevention requires the promotion of healthy lifestyle choices, such as reducing the consumption of sugar-sweetened beverages and ultra-processed foods, encouraging the intake of fresh and natural foods, and increasing physical activity from an early age. In terms of secondary prevention, it is essential to provide comprehensive care for individuals already diagnosed with T2DM. Existing prevention strategies must be continuously updated to ensure optimal disease management and glycemic control.

The observed differences in DALYs between men and women, as well as changes in these patterns over time, underscore the need for gendered public health strategies. The higher proportion of T2DM cases among women compared to men can be attributed to a multifactorial interaction of biological, sociodemographic, and behavioral determinants. From a pathophysiological standpoint, women are subject to conditions such as polycystic ovary syndrome (PCOS), menopause, and a history of gestational T2DM, all of which contribute to increased insulin resistance and heightened risk of developing the disease. Moreover, obesity prevalence is significantly higher among adult women in various population settings, representing a key risk factor [20]. Longer female life expectancy also increases cumulative exposure to metabolic risk factors. Additionally, women tend to have more frequent contact with healthcare services, facilitating earlier and more frequent diagnosis of T2DM, potentially leading to higher reported prevalence rates. In contrast, underdiagnosis may occur among men due to lower healthcare utilization [21].

High-income countries have made substantial progress in T2DM management through the strengthening of healthcare systems, resulting in reduced rates of complications and mortality. For example, the United States accounted for approximately 53% of global healthcare expenditure on diabetes, whereas countries such as India—despite having one of the largest populations of people with diabetes—invested less than 1% of total global spending [22]. These disparities highlight the critical need for adequate funding of diabetes prevention and management programs, particularly in low- and middle-income countries, to mitigate the burden and complications associated with T2DM. Furthermore, robust leadership and strong political commitment—supported by high-quality data—are essential for the continuous monitoring and evaluation of diabetes control strategies.

In this context, findings related to the state-level burden of T2DM offer valuable insight for informing health policy and guiding decisions aimed at improving disease management within specific regions. Nevertheless, it is important to emphasize that the cost of T2DM remains substantial, both in terms of human lives and economic impact.

This analysis leverages complete, population-based mortality and morbidity records for Tabasco (2013–2023), avoiding sampling bias and enabling robust join-point detection of temporal shifts. DALYs were computed with the GBD-2019 life table and disability weights, ensuring international comparability, and all input datasets plus calculation scripts are openly deposited, maximizing reproducibility and external auditability.

### Implications for the Health Care System

The predominance of premature mortality (YLL 84%) and the concentration of DALYs in adults older than 50 years point to the urgent need for earlier detection and control of cardiometabolic risk within primary care. Priorities include opportunistic systematic screening for diabetes and obesity starting at age 35 years; expansion of integrated chronic disease clinics in rural municipalities, where underreporting is highest; gender-specific education and self-management programs, given the double burden of DALYs for women; and routine linkage of mortality and morbidity databases to enable multicausal surveillance. Implementation of these measures could avert 30% to 35% of the DALYs projected for the next decade, based on the GBD population-attributable fractions for elevated BMI and hypertension.

## 5. Conclusions

T2DM represents a growing public health challenge in Mexico, characterized by a sustained increase in incidence and mortality. Between 2006 and 2019, the national incidence rate increased by approximately 35%, according to data from the Global Burden of Disease. This phenomenon is due to a complex interaction of factors, including population aging, genetic predisposition, changes in dietary patterns, and a prevalence of obesity above 75% in the adult population [20]. In the state of Tabasco, the situation is particularly critical: In 2021, the highest T2DM mortality rate in the country was recorded, highlighting structural failures in the coverage and quality of health services [2]. The COVID-19 pandemic exacerbated this problem, especially affecting people with comorbidities such as obesity, hypertension, and TDM2. Nearly 50% of the patients hospitalized for COVID-19 with severe evolution were diabetics, mostly between 50 and 65 years of age, which contributed to the increase in mortality since 2020 [23].

The burden of disease due to T2DM, measured by Disability Adjusted Life Years (DALYs), has shown significant growth in the last two decades, with notable peaks in 2016, 2020, 2021, and 2023. This increase coincides with a greater effect in the 50–64 and >65 age groups and a disproportionate burden on older women, reflecting inequalities in the impact of the disease. In addition, the economic impact is considerable for both the health system and households due to the high costs derived from complications and prolonged care. It is estimated that more than 65% of T2DM cases in people under 40 years of age remain undiagnosed, which shows critical weaknesses in the screening, primary care, and clinical follow-up systems [24,25,26]. Given this scenario, it is imperative to implement comprehensive and updated public policies that strengthen primary and secondary prevention, improve timely diagnosis, and promote adequate metabolic control. These strategies should be based on the promotion of healthy lifestyles, the strengthening of health infrastructure, and the guarantee of equitable access to health services, with special attention to the most vulnerable groups, particularly women and the elderly [27].

To comprehensively address the growing burden of T2DM in Mexico, the following strategic recommendations are proposed:

1. Allocation of resources: It is a priority to allocate specific funding, ideally from taxes on unhealthy products, to programs for the prevention of overweight and obesity, health education, and improved access to timely medical care.
2. Promoting scientific research: Inter-institutional research on the social and biological determinants of T2DM should be strengthened, as well as the evaluation of effective interventions in different socio-cultural contexts to improve its prevention, diagnosis, and treatment.
3. Evidence-based policy design: Interventions should be based on the best available scientific evidence, considering key aspects such as nutritional labeling, advertising regulation, and the promotion of healthy habits from early stages of life.
4. Prevention from infancy: It is essential to implement interventions that promote proper nutrition, physical activity, and breastfeeding from pregnancy and early childhood, with emphasis on supporting mothers and caregivers.
5. Continuous training of health personnel: Continuous training of primary care personnel should be guaranteed to improve early detection, comprehensive treatment, and effective follow-up of DM2, with a focus on education and nutritional control.
6. Reorientation of the model of care: A structural change is proposed towards a preventive model of care, focused on reducing risk factors throughout the life course and promoting healthy lifestyles.
7. Multidimensional strategies: The eradication of DM2 requires comprehensive approaches involving individuals, families, communities, and government sectors, including food, urban, and school policies that promote healthy environments.
8. Monitoring and evaluation: It is essential to implement robust evaluation systems to measure the effectiveness of policies and their impact on the prevalence, mortality, and burden associated with DM2.

These coordinated actions will significantly reduce the incidence and mortality from T2DM, improving the quality of life of the population and reducing its economic and social impact [20].

### Study Limitations

The absence of recent and substantial incidence and mortality data complicates the execution of a recent analysis. According to ENSANUT, Mexican adults with DM2 have at least one concomitant cardiometabolic disorder. Therefore, our estimates should be interpreted as a conservative minimum of the true burden. We recommend that future studies use linked databases or multicausal life table models to quantify this effect.

## Data Availability

Information on annual deaths was obtained from the General Directorate of Health Information (DGIS), through its public database for the period 2013–2023 [DGIS, http://www.dgis.salud.gob.mx/contenidos/basesdedatos/bdc_defunciones_gobmx.html] (accessed on 6 February 2025). Data on life expectancy were extracted from the National Institute of Statistics and Geography (INEGI), applying filters by state, year, and sex [https://www.inegi.org.mx/app/tabulados/interactivos/?pxq=Mortalidad_Mortalidad_09_1ac777ca-020d-4a8c-ae90-ec74f60b6ae8] (accessed on 11 March 2025). The incidence of the disease, disaggregated by age group and state, was obtained from the Morbidity Yearbook for the period 1984–2023, available on the official website of the General Directorate of Epidemiology [https://epidemiologia.salud.gob.mx/anuario/html/incidencia_enfermedad.html] (accessed on 17 April 2025).

## Supplementary Materials

The following supporting information can be downloaded at: https://www.mdpi.com/article/10.3390/ijerph22070997/s1, Sensitivity analysis (https://goo.su/2scxp, accessed on 30 April 2025). Database (https://goo.su/d4meq, accessed on 30 April 2025, https://goo.su/ogPka, accessed on 30 April 2025). Jointpoint (https://goo.su/VGDOPb, accessed on 30 April 2025).

## Abbreviations

The following abbreviations are used in this manuscript:

TDM2	Type 2 Diabetes Mellitus
DALYs	Disability-Adjusted Life Years
YLD	Years Lived with Disability
YLL	Years of life lost
