# Supplementary figures and images for: Estimation of the Burden of Disease Due to Diabetes Mellitus Type 2 in the Population of Tabasco During the Period 2013–2023
Source: Int J Environ Res Public Health. 2025 Jun 24;22(7):997. doi: 10.3390/ijerph22070997 (PMC12294181; doi:10.3390/ijerph22070997)

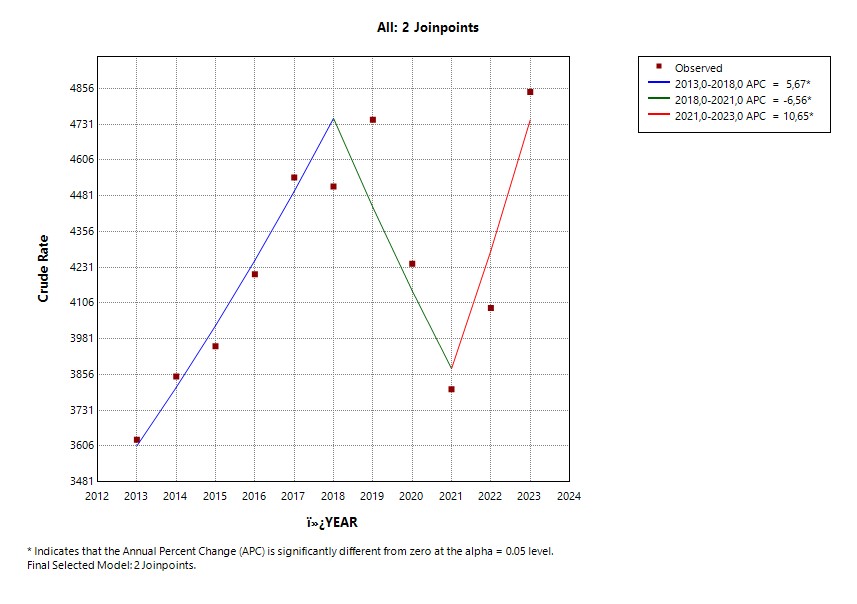

Supplement: Supplementary file 1 [file ijerph-22-00997-s001.zip › S3-jointpoint.jpeg]
